# Unintentional drug-related deaths in people with mental illness in NSW Australia, 2012–2016: a retrospective cohort study

**DOI:** 10.1007/s00127-022-02280-4

**Published:** 2022-05-03

**Authors:** Jennifer Smith-Merry, Kenji Fujita, Tim Chen, Andrew Baillie

**Affiliations:** 1 Centre for Disability Research and Policy, School of Health Sciences, Faculty of Medicine and Health, The University of Sydney, Sydney, NSW 2006 Australia; 2 School of Pharmacy, Faculty of Medicine and Health, The University of Sydney, Sydney, NSW 2006 Australia; 3 School of Health Sciences and Matilda Centre for Research in Mental Health and Substance Abuse, Faculty of Medicine and Health, The University of Sydney, Sydney, NSW 2006 Australia

**Keywords:** Mortality, Pharmaceutical drugs, Illicit drugs, Drug interactions, Adverse events, Mental illness

## Abstract

**Purpose:**

People with mental illness are a vulnerable and stigmatised group with poor health outcomes including greater premature mortality. This study aimed to investigate trends and rates of change in unintentional drug-related deaths for people with mental illness, describe types of medicines involved, and identify populations at risk in a cohort from New South Wales, Australia.

**Methods:**

Features of unintentional drug-related deaths for people with mental illness between 2012 and 2016 were identified in a retrospective review of data from the National Coronial Information System.

**Results:**

A total of 495 unintentional drug-related deaths were identified (1.6 deaths/100,000 population), showing an upward trend (*p *< 0.01). The most common substance involved was diazepam in both genders (males 135/319, 42%, female 76/176, 43%) and more than one contributory drug was included in 80% of cases. Between 2012 and 2016, amphetamine-related deaths showed the highest increase (3.2-fold), followed by codeine (2.5-fold) and quetiapine (2.5-fold). Males (RR 1.8, 95% CI 1.5–2.2) and people aged 35–44 (RR 1.7, CI 1.3–2.2) were more likely to die from unintentional drug-related deaths compared with the reference (females and people aged 25–34).

**Conclusion:**

This study found that the drugs commonly involved in deaths are also the drugs commonly used by and prescribed to people with mental illness. There were also significant differences between gender, age group, and marital status in the trend and rate of unintentional drug-related deaths for people with mental illness. A multifaceted approach encompassing both pharmaceutical prescribing and targeted public health messaging is required to inform intervention and prevention strategies.

**Supplementary Information:**

The online version contains supplementary material available at 10.1007/s00127-022-02280-4.

## Introduction

The number of unintentional drug-related deaths has increased significantly over past decades, representing a significant public health problem worldwide. Deaths due to unintentional drug-related poisoning exceed road traffic crash deaths in the United States (US), the United Kingdom (UK), and Australia [1, 2]. Between 2001 and 2017, the number of unintentional drug-related deaths in Australia rose by 64%—from 981 to 1612 deaths, accounting for almost three quarters (75%) of all drug-related deaths [2]. Amongst these deaths, opioids were the most commonly identified drug group, followed by benzodiazepines and stimulants [2]. In addition, alcohol and poly-drug use contributed significantly to these deaths. Overdose from opioids, illicit drugs, alcohol, and poly-drug causes has a strong association with mental disorder [3, 4]. People with schizophrenia, bipolar disorder, schizoaffective disorder, and major depressive disorder have an excess mortality from these causes, being two or three times as high as that in the general population [5–7]. Accordingly, Australian policy responses to both mental illness and drug and alcohol use (e.g., the *National Drug Strategy*) have included co-morbid mental illness and drugs and alcohol use as a core focus area.

Mental illness is common in Australia, with around 1 in 5 people aged 16–85 experiencing a mental illness in any year [8]. An estimated 3.3% of Australian adults experience a severe mental illness each year, of whom one-third (1.1% of adults) experience a persistent mental illness that requires ongoing services to address residual disability [9, 10]. In addition, 71% of Australians who receive a mental health support have difficulty with drug and alcohol use [11]. Compared with people without mental illness, people with mental illness are about 1.2 times as likely to drink alcohol at levels that exceed the lifetime risk (defined as consuming no more than 10 standard drinks per week on average; and not consuming more than 4 standard drinks on a single day in each month) (21% compared with 17%), 1.7 times as likely to have recently used any illicit drug (26% compared with 15%), 2.1 times as likely to use pharmaceuticals for non‑medical purposes (7.6% compared with 3.6%) [12], and twice as likely to use prescribed opioids [13]. Moreover, a previous study has shown that a large proportion of people who died due to prescription drug overdose had histories of mental illness [14]. In 2011, mental and substance-use disorders were responsible for an estimated 12% of the total disease burden in Australia, making it the third highest group of diseases behind cancer and cardiovascular diseases [15].

Despite the increase in unintentional drug-related deaths and the association between these deaths and mental illness, few studies have evaluated this relationship in this cohort of Australians. Limited information on unintentional drug-related deaths for people in this group impedes policy design, implementation, and evaluation of prevention strategies. This gap is particularly problematic given that this is a group that are already vulnerable because of unmet and often complex needs [16] are socially stigmatised [17] and therefore experience considerable social marginalisation [18, 19]. In relation to this gap, the aims of this study were to use a mortality cohort from New South Wales (NSW), Australia, to investigate the trends and rate of changes in unintentional drug-related deaths for people with mental illness, describe types of medicines involved, and identify populations at risk. NSW is Australia’s most populous state, with a population of over 8 million in 2021, two-thirds of whom live in the capital city Sydney [20].

## Methods

A retrospective examination of NSW mortality records in the National Coronial Information System (NCIS) between 1 January 2012 to 31 December 2016 was conducted.

### Data sources

The NCIS is a national Internet-based storage and retrieval system for deaths that are notified to a Coroner. All deaths not presumed to have a natural cause must be notified to a Coroner in Australia. The NCIS is a medico-legal database and contains demographic information on the deceased and data on the circumstances of death along with various legal and medical documents (i.e., autopsy, toxicology, coronial findings, and/or police reports). In this study, with assistance from the NCIS access liaison officer, NSW coronial case records pertaining to unintentional drug-related deaths for persons with mental illness were extracted (further details in ‘case selection’ below). Only deaths marked ‘unintentional’ were included in the analysis for this paper because of the different circumstances surrounding drug-related intentional deaths.

Except in a very limited range of cases, the NCIS does not record as one of its standard categories whether a person has had a mental illness at the time of their death. Accordingly, a methodological framework was devised to identify relevant death records based on testing of multiple search terms to extract relevant records via text-searching of documents included in each file. The finalised search strategies were developed using an iterative process during which death records identified from individual search terms were screened for relevance by reading through each file. Records were deemed not to be relevant if there was no reference to mental illness. For example, we removed ‘mental health’ which was a standard wording on police forms, and ‘depressed’ as a search term because of the high number of irrelevant results obtained coming from the autopsy report about depressed scars or regions of anatomy. However, most of the small number of relevant files found with these search terms were identified using other search terms (e.g., mental illness). Through this process, we determined a matrix of search terms which would maximise identification of relevant records but also avoid high numbers of irrelevant files (and ethical concerns about unnecessary access to personally sensitive records). Based on this process, we calculated that the final search strategy missed only 3.4% of relevant results. The exact search strategies are included in online supplementary table S1. Of the 1456 deaths in people with mental illness over the 2012–2016 period identified through this strategy, 495 were attributed by the Coroner to a primary drug-related cause. In comparison, the number of deaths referred to the coroner in NSW for the 2012–2016 period was 24,770 and the number of drug-related deaths in NSW for all groups (i.e., not just those with mental illness) was 2647 [21, 22].

### Case selection

#### Inclusion criteria

Cases were included if they fulfilled the following criteria: (A) the intent of death was found by a Coroner to be unintentional, (B) individuals have had interactions with the mental health system prior to their death (community mental health or mental health-related inpatient or outpatient service use), or the person was noted as having a significant and ongoing mental illness in the documents relating to their death. In accordance with the NSW Mental Health Act, which applies to this data collection context, mental ill-health was defined as “a condition that seriously impairs, either temporarily or permanently, the mental functioning of a person and is characterised by the presence in the person of any one or more of the following symptoms: (a) delusions, (b) hallucinations, (c) serious disorder of thought form, (d) a severe disturbance of mood, and (e) sustained or repeated irrational behaviour indicating presence of any one or more of the symptoms [23]. Importantly for the analysis presented here, we did not classify drug or alcohol dependence or use as a mental health disorder in and of itself.

#### Exclusion criteria

Cases were excluded if the coronial cases were not finalised by a Coroner (i.e., cases remain open or under investigation).

### Data extraction and management

Data regarding age, gender, marital status, year of death, and substances contributing to death were extracted from the retrieved cases. Substances contributing to the cause of death were extracted primarily from the electronically registered information categorised as a primary or secondary cause of death. Each single drug extracted was classified using the Anatomical Therapeutic Chemical (ATC) classification of the World Health Organization. When no substances were registered, the other reports (i.e., autopsy, toxicology, and police report) were referenced. In addition, population data by age and gender in each year during the study time period was sourced from the Australian Bureau of Statistics (ABS) [24]. The age group 0 to 14 years was disregarded, because no death case was found. Proportion of marital status by age and gender in 2016 was used to estimate population by age, gender, and marital status between 2012 and 2015 [25]. Python 3.6.5 was used for data pre-processing and transformation.

### Analysis

Descriptive statistics were computed for the results of the present study based on counts and proportions where relevant. Welch’s test was used for assessing the mean age differences between male and female, because Levene’s test for homogeneity of variances indicated unequal variances between the groups. Cochran–Armitage test was used to evaluate trends in mortality rate over time. Due to over-dispersion, negative binomial regression analyses using the number of deaths as the dependent variable and sex, age group, marital status, and year as independent variables with an offset of the log of the population were conducted to calculate the rate ratio (RR) and their corresponding 95% confidence intervals (CI) for each independent variable. Female was used as the reference, because the prevalence of mental illness is higher in females compared with males [26]. The age group of 25–34 years were used as the reference because of its historical prominence as a high-risk group for unintentional drug-related deaths in general [27]. The Akaike information criterion (AIC) was used as a model selection criterion [28]. All statistical analyses were performed using the statistical package R version 3.6.1. For negative binomial modelling, we used the glm.nb function in the MASS package.

## Results

During 2012–2016, a total of 495 cases were identified as unintentional drug-related deaths for people with mental illness (1.6/100,000 population, Table 1). Crude mortality rates in 2016 (2.0/100,000 population) were 40% higher than those in 2012 (1.4/100,000), showing a statistically significant trend (*p *< 0.01).

**Table 1:** Unintentional drug-related deaths for people with mental illness by demographic characteristics in NSW, Australia, 2012–2016

	2012^a^	2013^a^	2014^a^	2015^a^	2016^a^	Total^a^	Fold increase (2012 VS 2016)	Trend^b^ *χ*^2^	Rate ratio (CI)	*p* value
Gender										
Male	50 (1.7)	55 (1.9)	61 (2.0)	72 (2.4)	81 (2.6)	319 (2.1)	1.5	7.7**	1.81 (1.48–2.22)	< .001
Female	34 (1.1)	26 (0.9)	30 (1.0)	40 (1.3)	46 (1.4)	176 (1.1)	1.3	3.1	1*	
Age (years)										
15–24	2 (0.2)	3 (0.3)	1 (0.1)	6 (0.6)	9 (0.9)	21 (0.4)	4.4	6.6*	0.16 (0.01–0.26)	< .001
25–34	17 (1.6)	26 (2.4)	23 (2.1)	27 (2.4)	23 (2.0)	116 (2.1)	1.2	0.2	1*	
35–44	25 (2.5)	20 (2.0)	30 (2.9)	42 (4.1)	35 (3.4)	152 (3.0)	1.4	5.4*	1.71 (1.31–2.24)	< .001
45–54	24 (2.4)	18 (1.8)	28 (2.8)	19 (1.9)	39 (3.9)	128 (2.6)	1.6	3.6	1.29 (0.96–1.73)	0.09
55–64	10 (1.2)	10 (1.2)	5 (0.6)	14 (1.6)	20 (2.2)	59 (1.4)	1.9	4.1*	0.73 (0.50–1.05)	0.09
≥ 65	6 (0.6)	4 (0.4)	4 (0.3)	4 (0.3)	1 (0.1)	19 (0.3)	0.1	3.2	0.17 (0.09–0.30)	< .001
Marital status										
Married/de facto	23 (0.8)	17 (0.6)	22 (0.7)	21 (0.7)	33 (1.1)	116 (0.8)	1.4	1.8	1*	
Never married	32 (1.5)	36 (1.7)	36 (1.7)	52 (2.4)	51 (2.3)	207 (1.9)	1.5	5.7*	3.25 (2.54–4.16)	< .001
Divorced	12 (2.5)	11 (2.2)	11 (2.2)	9 (1.8)	14 (2.7)	57 (2.3)	1.1	0.0	3.36 (2.41–4.62)	< .001
Separated	4 (2.2)	4 (2.1)	8 (4.2)	10 (5.2)	7 (3.6)	33 (3.5)	1.7	1.9	4.32 (2.88–6.29)	< .001
Widowed	4 (1.3)	2 (0.7)	2 (0.6)	5 (1.5)	1 (0.3)	14 (0.9)	0.2	0.5	4.29 (2.24–7.65)	< .001
Unlikely to be known	9 (N/A)	11 (N/A)	12 (N/A)	15 (N/A)	21 (N/A)	68 (N/A)	N/A	NA	NA^c^	NA^c^
Total	84 (1.4)	81 (1.3)	91 (1.5)	112 (1.8)	127 (2.0)	495 (1.6)	1.4	10.7**		

^a^Number of deaths (mortality rate per 100,000)

^b^*p < 0.05, **p < 0.01, ***p < 0.001

^c^68 cases (unlikely to be known) excluded

**Fig. 1:**
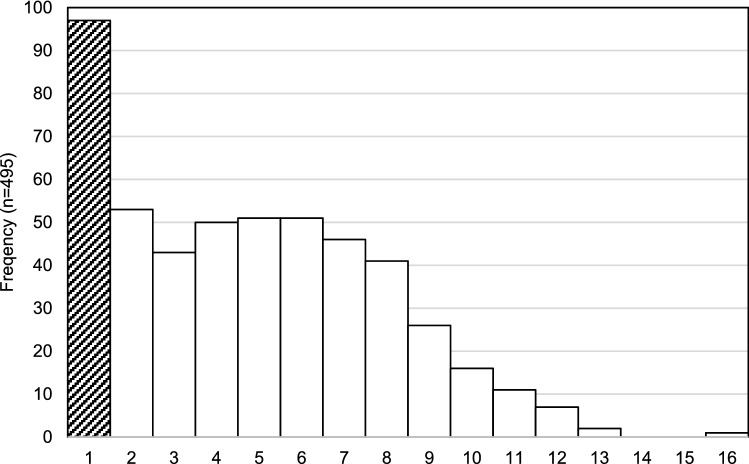
The number of drugs involved per deceased individual

**Fig. 2:**
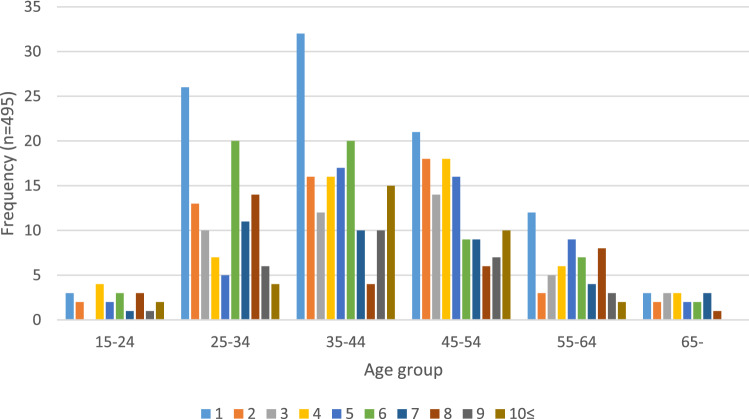
The number of drugs involved per deceased individual by age group

Drug toxicity may be caused by multiple factors including dosage and interactions between multiple prescribed and unprescribed drugs. The median number of drugs involved per case was 5.0 (IQR 2.0–7.0) with the range being 1 to 16 individual drugs identified as being implicated in the individual’s death. The most frequent number of drugs involved (i.e., mode) was 1 (97/495 cases, 20%, Fig. 1). Young men aged 25–34 and 35–44 years were most likely to die with only one drug involved (Fig. 2). Amongst the single drug deaths, heroin (ATC code: N02AA09) and methadone (N07BC02) were the most common drugs (21/97 and 10/97, respectively, Table 2). This was different from drugs involved in multiple drugs deaths. As shown in Table 3, the most common substance involved amongst the 495 cases was diazepam (N05BA01) in both genders (males 135/319, 42%, females 76/176, 43%). This was followed by temazepam (males 86/319, 27%, females 46/176, 26%), codeine (males 79/319, 25%, females 51/176, 29%), morphine (males 83/319, 26%, females 41/176, 23%), oxazepam (males 73/319, 23%, females 39/176, 22%), oxycodone (males 62/319, 19%, females 46/176, 26%), paracetamol (males 51/319, 16%, females 52/176, 29%), alprazolam (males 63/319, 20%, females 30/176, 17%), methadone (males 64/319, 20%, females 28/176, 16%), and quetiapine (males 51/319, 16%, females 40/176, 23%). Alcohol and heroin (N02AA09) were predominantly involved in deaths amongst males (both *p *< 0.05), whilst paracetamol (N02BE01) and amitriptyline (N06AA09) were more common in females (both *p *< 0.001). Between 2012 and 2016, amphetamine (N06BA01) showed the highest increase (3.2-fold), followed by codeine (R05DA04, 2.5-fold), quetiapine (N05AH04, 2.5-fold), and morphine (N02AA01, 2.2-fold).

**Table 2:** Drugs involved in a death from only a single drug

Name	ATC code	Number (%)
Heroin	N02AA09	21 (22)
Methadone	N07BC02	10 (10)
Morphine	N02AA01	7 (7)
Oxycodone	N02AA05	7 (7)
Amphetamine	N06BA01	7 (7)
Fentanyl	N02AB03	7 (7)
Clozapine	N05AH02	7 (7)
Quetiapine	N05AH04	3 (3)
Olanzapine	N05AH03	2 (2)
Citalopram	N06AB04	2 (2)
Others		24 (25)
Total		97 (100)

**Table 3:** Individual drugs involved in unintentional drug-related deaths for people with mental illness in NSW, Australia, 2012–2016

Substance	ATC code	Total (*n *= 495)		Fold increase (2012 VS 2016)	Male (*n *= 319)		Female (*n *= 176)		Male versus female^b^ *χ*^2^
		Number of cases^a^	%		Number of cases^a^	%	Number of cases^a^	%	
Diazepam	N05BA01	211	42.6	1.5	135	42.3	76	43.2	0.008
Alcohol	N/A	159	32.1	1.9	113	35.4	46	26.1	4.1*
Temazepam	N05CD07	132	26.7	1.5	86	27.0	46	26.1	0.01
Codeine	R05DA04	130	26.3	2.5	79	24.8	51	29.0	0.8
Morphine	N02AA01	124	25.1	2.2	83	26.0	41	23.3	0.3
Oxazepam	N05BA04	112	22.6	1.9	73	22.9	39	22.2	0.01
Oxycodone	N02AA05	108	21.8	1.0	62	19.4	46	26.1	2.6
Paracetamol	N02BE01	103	20.8	1.7	51	16.0	52	29.5	11.8***
Alprazolam	N05BA12	93	18.8	1.6	63	19.7	30	17.0	0.4
Methadone	N07BC02	92	18.6	1.2	64	20.1	28	15.9	1.0
Quetiapine	N05AH04	91	18.4	2.5	51	16.0	40	22.7	3.0
Amphetamine	N06BA01	61	12.3	3.2	46	14.4	15	8.5	3.1
Heroin	N02AA09	50	10.1	2.0	40	12.5	10	5.7	5.1*
Amitriptyline	N06AA09	49	9.9	1.3	18	5.6	31	17.6	16.9***
Olanzapine	N05AH03	47	9.5	1.9	30	9.4	17	9.7	0.0

^a^A total of 2323 substances were identified amongst 495 deceased. Because more than 1 substance could be involved par case, frequencies for substances total > 100%

^b^*p < 0.05, **p < 0.01, ***p < 0.001

Of the 495 cases, 319 (64%) were male and 176 (36%) females. The mean age of females was 46.9 years, 6.3 years higher than that of males (40.6 years, *p *< 0.001). Although the mortality rate was higher in 2016 than in 2012 for both genders, only the rate for males showed a significant upward trend (*p *< 0.01). In NSW between 2012 and 2016, males with mental illness were 1.8 times (CI 1.5–2.2) more likely than females to die from unintentional drug-related causes (Table 1). In terms of the age categories, the highest mortality rate was those aged 35–44 years (3.0/100,000 population), showing a significant upwards trend (*p *< 0.05). Using the age group of 25–34 years as the reference, people aged 35–44 years were 1.7 times (CI 1.3–2.2) more likely to die from the unintentional drug-related deaths. Meanwhile, the youngest (15–24 years) and oldest (≥ 65 years) age groups had lower adjusted rate (RR 0.16, CI 0.01–0.3 and RR 0.17, CI 0.1–0.3 respectively). In terms of the marital status groups, when using people with mental illness who were married/de facto as the reference, the rate of dying from unintentional drug-related deaths was more than three times higher in all other groups (i.e., never married, divorced, separated, and widowed) than the reference. Those who separated had the highest mortality rate (3.5/100,000) and likelihood of dying (RR 4.3, CI 2.9–6.3), whilst those who were married/de facto had the lowest rate (0.8/100,000).

## Discussion

This study revealed that 495 people with mental illness died of unintentional drug-related causes in NSW between 2012 and 2016, representing 18.7% of all drug-related deaths in the state. Unintentional drug-related deaths in people with mental illness increased over the period. A better understanding of the types of drugs and demographic profile of people with mental illness who die from unintentional drug-related deaths will help develop more targeted intervention and prevention efforts.

In Australia, anxiety disorders (for example, post-traumatic stress disorder, and social phobia) are common, affecting 14% of adults, followed by affective disorders (for example, depression, 6%) [29]. They frequently co-exist with severe and continuing mental disorders and substance misuse [30]. Benzodiazepines are central nervous system depressants that are commonly prescribed to treat insomnia, stress, or anxiety. From 2010 to 2015, the main forms of benzodiazepines that were dispensed in Australia were diazepam, temazepam, and oxazepam [31]. Amongst the 495 deaths identified in this study, benzodiazepine derivatives, such as diazepam (*n *= 211), temazepam (*n *= 132), oxazepam (*n *= 112), and alprazolam (*n *= 93), were involved in the largest number of deaths. Of the 97 single-drug deaths; however, only 1 death was due to a benzodiazepine derivative (i.e., alprazolam). This implies that fatal overdose is less likely with a single benzodiazepine derivative than with a single opioid analgesic such as heroin (*n *= 21), morphine (*n *= 7), oxycodone (*n *= 7), and fentanyl (*n *= 7). This is consistent with previous research which shows that benzodiazepines are frequently involved in fatal and non-fatal overdoses in Australia and elsewhere [31–33]. Benzodiazepines are an important medication in the treatment of mental illness, but there are a range of negative outcomes which may result from poor prescribing practices around their use, including long-term unmonitored usage [34]. The data presented here provide additional impetus for continuing efforts to improve benzodiazepine prescribing. However, analysis of previous efforts to change practice shows that an approach that only targets demand-side factors such as cost do not adequately address poor prescribing practices and that strategies need to be targeted at communities where there is problematic usage rather than the whole treatment population [35].

The results in this study further point towards the importance of broader efforts around prescribing awareness and medication monitoring practices, as well as medicines-related education for people with mental illness [36]. The number of overall deaths (*n *= 398, 80%) involving polypharmacy or interactions between illicit, over the counter and prescribed medications which increase the risk of drug interactions was high. Our results reflect broader concerns about polypharmacy in the context of mental illness [37, 38] and the necessity for enhanced prescribing monitoring strategies for this group. Our results show that such strategies should also be connected with mental health and drug and alcohol strategies. Tackling each of these separately will not allow us to address the interdependence of these elements in the deaths of people with mental illness. The challenge for healthcare systems will be how to establish integrated mechanisms that enable real-time identification of people at high risk of drug-related death for further assessment, and prioritisation for specialised multidisciplinary support.

People who were male, aged 35–44, never married, divorced, separated, and widowed were more likely to die from unintentional drug-related deaths. There was an upward trend in unintentional drug-related deaths for people with mental illness between 2012 and 2016 which was more pronounced amongst the following parts of the community: males (1.5-fold), people aged 15–24 (4.4-fold), 35–44 (1.4-fold) and 54–64 (1.9-fold), and people who never married (1.5-fold). Previous Australian reports have found that males and people aged 30–59 years show an increase in the number of unintentional drug-induced deaths in general (not specific to people with mental illness) (1.4-fold and 2.1-fold, respectively), whilst those aged under 30 years showed a decrease (0.6-fold) between 2012 and 2017 [2]. Therefore, people aged 15–24 with mental illness may be a specific group in need of increased policy and practice attention. Although the actual number of deaths for this age group was small (*n *= 21), given this age group was most likely to experience very high levels of psychological distress amongst all age groups [8], specific mental health support may be required for them. It is imperative that all mental health and drug and alcohol services are able to provide services to people with co-morbid needs across both areas. However, this is a known and as yet still unresolved area of need in Australia.

Being married or in a de-facto relationship generally results in better mental and physical health outcomes compared to those who are divorced, separated, or widowed. Marital status has previously been found to be connected to social isolation and risk of suicide for those who are widowed, separated, divorced, or single [39]. Relationship breakdown and separation is also associated with increased risk of mental illness, alcohol abuse, and family violence [40, 41]. This study identified the large gap in rate ratios between people with mental illness who were married or in de-facto relationships and others. People with mental illness who had never married had the highest number of identified deaths (*n *= 207) and this showed an upward trend during the study period. This emphasizes the vulnerability of people with mental illness who are socially isolated and the need for efforts to prevent drug-related deaths to consider the impact of social isolation as an additional and compounding form of vulnerability [16, 42].

Importantly, although the prevalence of mental illness has been higher in females than in males for all age groups [26], males with mental illness were 1.8 times more likely than females to die from unintentional drug-related deaths. The types of mental illness people develop varies by gender, which might impact the types of drugs used and potential for overdose. For example, depression was more prevalent in females than in males [43]. Males are more likely to have substance-use disorders (e.g., alcohol and other drugs such as cannabis, heroin, and cocaine) in combination with either anxiety or affective disorders [44, 45]. These previous findings are consistent with our results that show unintentional drug-related deaths for males with mental illness as more likely to involve alcohol and heroin compared with those for females. Moreover, there are social and environmental factors that could delay males from seeking mental health assistance longer than females. Males are generally more reluctant to seek help as this may be seen as a threat to their masculinity because of the impact of stigma [46]. Further research is warranted to test these associations.

Uses of illicit drugs and medicines for non-medical purposes are common amongst people with mental illness. In 2019, compared to people with no mental illness, people with mental illness were 1.7 times as likely to have used any illicit drug in the previous 12 months and about 2 times as likely to have used meth/amphetamines and pharmaceuticals for non-medical reasons [10]. Opioid analgesics are commonly prescribed for pain management, and the treatment of heroin and other opioid dependence. These opioids are also some of the most commonly used pharmaceuticals for non-medical purposes. Given that the increase of the number of deaths involving amphetamine, codeine, and morphine was relatively higher between 2012 and 2016, more attention should be paid to these substances within the new and existing strategies referred to in this discussion.

### Strengths and limitations

Our review has some notable strengths. To our best knowledge, this is the first report regarding unintentional drug-related deaths for people with mental illness in Australia. Focusing the target population to people with mental illness highlighted the risk factors specific to this vulnerable population. We do however acknowledge that our approach has some limitations. During the screening process, we identified the relevant deaths by text-based searching of coronial documents and including cases based on our own assessment of the data included in these documents. Documents are not universally included for all cases and relied on the information included by the pathologist, coroner, and police in their reports. This means that the data here are likely an underestimation of the true extent of the issues discussed. Findings from this study should be compared in further studies using other existing and developing datasets, such as the NSW Death Screening and Database Project. Coronial judgements generally under-estimate intentional deaths, so some of the deaths included in this data may actually be intentional [47]. Because the rate of people with mental illness and levels of pharmaceutical use for non-medical purposes may vary depending on the patients’ usual area of residence [15, 31], findings identified in this study might not be applicable to other states or countries. This study should be replicated in other states and territories to investigate unintentional drug-related deaths in the setting of mental illness across Australia.

## Conclusion

In this study, we identified unintentional drug-related deaths in people with mental illness. The data showed that for most people, more than one drug contributed to their death and amongst the most commonly reported drugs were those associated with the treatment of mental illness. This highlights the urgent need to renew efforts to address polypharmacy and prescribing practices, particularly those in relation to benzodiazepines. These efforts must focus on the social and demographic context of people with mental illness rather than prescribing practices on their own. There were significant differences between gender, age group and marital status in the trend and rate of unintentional drug-related deaths for people with mental illness during 2012 and 2016 in NSW, Australia. The differences in the demographic characteristics of age, gender, and marital status imply that a disease- and demographic-specific multifaceted approach is required to reduce unintentional drug-related deaths for people with mental illness. Overall, our results demonstrate the vital importance of bringing policy approaches to mental illness together with those relating to drug and alcohol use and pharmaceutical prescribing and monitoring.

## Supplementary Information

Below is the link to the electronic supplementary material.

Supplementary file1 (DOCX 26 KB)

Supplementary file2 (DOCX 32 KB)

## Data Availability

Individual participant data are not available because of privacy considerations and ethical approval restrictions which mean that only aggregated or non-re-identifiable data can be reported. Full study protocol will be made freely available with publication via our institutional website.

## Code availability

Python 3.6.5 was used for data pre-processing and transformation. The Akaike information criterion (AIC) was used as a model selection criterion. All statistical analyses were performed using the statistical package R version 3.6.1. For negative binomial modelling, we used the glm.nb function in the MASS package.
