# Supplementary material for: Unintentional drug-related deaths in people with mental illness in NSW Australia, 2012–2016: a retrospective cohort study
Source: Soc Psychiatry Psychiatr Epidemiol. 2022 May 3;58(2):239–48. doi: 10.1007/s00127-022-02280-4 (PMC9922235; doi:10.1007/s00127-022-02280-4)
Supplement: Supplementary file 1 — Supplementary file1 (DOCX 26 KB) [file 127_2022_2280_MOESM1_ESM.docx]

Supplementary table 1: Search terms trialled and used to search the NCIS database

| Search terms used | Included in search – yes/no (with reason) |
| --- | --- |
| Anorexi* | Yes |
| Anxiety | Yes |
| Bipolar | Yes |
| Bulimia | Yes |
| “Mental Health Facility” | Yes |
| “Mental Health Team” | Yes |
| “Mental Health Unit” | Yes |
| “Mental Illness” | Yes |
| Psychosis | Yes |
| Psychotic | Yes |
| Schiz* | Yes |
| Depressed | No (high degree of irrelevancy) |
| Depression | No (picked up standard wording on police form) |
| Psychiatric | No (picked up standard wording on police form) |
| Mental | No (picked up standard wording on police form) |
